# Olfactory and taste dysfunction in coronavirus disease 2019 patients: its prevalence and outcomes

**DOI:** 10.1017/S0022215120002467

**Published:** 2020-11-16

**Authors:** A Jain, L Kumar, J Kaur, T Baisla, P Goyal, A K Pandey, A Das, L Parashar

**Affiliations:** 1 Department of ENT, Employees’ State Insurance Corporation (‘ESIC’) Medical College and Hospital, Faridabad, India; 2 Department of Community Medicine, Employees’ State Insurance Corporation (‘ESIC’) Medical College and Hospital, Faridabad, India; 3 Department of Physiology, Employees’ State Insurance Corporation (‘ESIC’) Medical College and Hospital, Faridabad, India

**Keywords:** Anosmia, Coronavirus, COVID-19, Dysgeusia, Smell

## Abstract

**Objectives:**

To evaluate the occurrence, clinical course and outcomes of olfactory and gustatory dysfunction in patients with laboratory confirmed coronavirus disease 2019 infection.

**Methods:**

This is a prospective cross-sectional study of patients diagnosed with coronavirus disease 2019 infection by reverse transcription polymerase chain reaction over two months. The epidemiological and clinical outcomes studied were: age, sex, general symptoms, and olfactory and taste dysfunction.

**Results:**

A total of 410 coronavirus disease 2019 infected patients were included in the study, with 262 males (63.9 per cent) and 148 females (36.1 per cent). Ninety-nine patients (24.1 per cent) reported chemosensory dysfunction, of which 85 patients (20.7 per cent) reported both olfactory and taste dysfunction. Olfactory and taste dysfunction were proportionally more common in females. The mean duration of olfactory and taste dysfunction was 4.9 days, with a range of 2–15 days.

**Conclusion:**

Olfactory and taste dysfunction are prevalent symptoms in coronavirus disease 2019 patients. In this study, they were more common in females than males. The occurrence of such dysfunctions is lower in the Indian population than in the European population.

## Introduction

A novel severe acute respiratory syndrome coronavirus-2 (SARS-CoV-2) infection, coronavirus disease 2019 (Covid-19), emerged in East Asia at the end of 2019, and, since then, it has rapidly spread to the rest of the world.^1^ The clinical manifestations of Covid-19 infection vary widely, ranging from no symptoms (an asymptomatic carrier) to severe acute respiratory distress syndrome and death. The most common symptoms include fever, cough, dyspnoea, sore throat, headache, myalgia, rhinorrhoea and diarrhoea.^2,3^ While patients with a known travel history, previous contact with a positive patient or characteristic symptoms may easily be identified, patients with non-classical symptoms may be missed and may serve as vectors of transmission of the disease, leading to its further spread in the population.

The spread of Covid-19 throughout the world has highlighted chemosensory dysfunction as a symptom of the disease. Smell and taste dysfunction during Covid-19 infection have been reported as uncommon in China.^4^ Whereas, Europe has reported a very high frequency of chemosensory dysfunction in Covid-19 infected patients.^5–8^ Smell and taste dysfunction may appear in the initial stages of viral infection and in asymptomatic patients.^5–7^ Such dysfunction may therefore assist in diagnosing asymptomatic Covid-19 infected individuals and thus contribute to limiting the spread of this disease.

This study aimed to evaluate and characterise the occurrence, clinical course and outcomes of olfactory and taste dysfunction in patients with laboratory confirmed Covid-19 infection. At present, there are no published data regarding the frequency of olfactory and gustatory dysfunction in Covid-19 infected patients in the Indian population.

## Materials and methods

This study was carried out at the Employees’ State Insurance Corporation (‘ESIC’) Medical College and Hospital, Faridabad, India, from May 2020 to June 2020 (two months). This study was approved by the Institutional Ethics Committee. The patients who tested positive for Covid-19 infection based on reverse transcription polymerase chain reaction findings at our hospital during this period were identified and contacted. The patients with mild to moderate disease and a willingness to participate in the study were included. The exclusion criteria were: children (less than 18 years of age), or patients with psychiatric or neurological disorders, a history of previous surgery or radiation in the oral and nasal cavities, chronic rhinosinusitis, pre-existing smell and taste disturbance, and those on assisted ventilation.

This was a prospective, cross-sectional study of patients diagnosed with Covid-19 infection. The data were collected during ENT consultation or over the telephone. Patients who could not be contacted even after three attempts were not included in the study.

Demographic characteristics of the patients included in the study were recorded. A detailed clinical history of all the patients was taken, including the onset and duration of symptoms. History regarding olfactory and taste dysfunction was documented. The order in which symptoms appeared, and details of whether olfactory and taste dysfunction appeared before or after other symptoms, were documented.

All patients were asked to rate their sense of smell at its worst point during the infection, as normal, decreased (hyposmia) or none at all (anosmia). They were also asked if they had any alteration of taste sensation (dysgeusia). In a follow-up telephone call, the duration of olfactory and taste dysfunction was documented. The patients were asked whether their olfactory and taste dysfunction persisted even after discharge from the hospital or after a laboratory confirmed Covid-19 negative report. The patients were followed up until their loss of smell or taste recovered.

Data were collected and entered into Microsoft Excel® spreadsheet software. Data analysis was conducted using SPSS® statistical software version 25. The results were explained in terms of simple percentages. For qualitative data, differences between groups were assessed using the chi-square test. A *p*-value less than 0.05 was considered statistically significant.

## Results

A total of 410 Covid-19 infected patients were included in the study. There were 262 males (63.9 per cent) and 148 females (36.1 per cent). The age range was 18–77 years, with a mean of 37.21 ± 13.30 years.

The patients’ general symptoms during the infection were: malaise or weakness, in 211 patients (51.5 per cent); sore throat, in 96 (23.4 per cent); cough, in 99 (24.1 per cent); fever, in 146 (35.6 per cent); and nasal obstruction or discharge, in 40 (9.7 per cent).

Ninety-nine patients (24.1 per cent) reported chemosensory dysfunction, of which 85 (20.7 per cent) reported both olfactory and taste dysfunction, 92 (22.4 per cent) reported olfactory dysfunction and 92 (22.4 per cent) reported taste dysfunction. Among the 92 patients (22.4 per cent) with olfactory dysfunction, 66 (16.1 per cent) had hyposmia and 26 (6.3 per cent) had anosmia. Olfactory dysfunction was proportionally more common in females than males (Table 1), and this difference was statistically significant (*p* < 0.05). Taste dysfunction was also proportionally more common in females, but this difference was not statistically significant. Overall, olfactory or taste dysfunction was proportionally more common in females, and this difference was statistically significant (*p* < 0.05).

**Table 1.** Demographic characteristics and olfactory and taste dysfunction

	Olfactory dysfunction		Taste dysfunction		Olfactory or taste dysfunction	
Characteristic	No (*n* = 318)	Yes (*n* = 92)	No (*n* = 318)	Yes (*n* = 92)	No (*n* = 311)	Yes (*n* = 99)
Age						
– <50 years	260	79	260	79	253	86
– >50 years	58	13	58	13	58	13
– *p*-value	0.359		0.359		0.206	
Sex						
– Male	212	50	211	51	208	54
– Female	106	42	107	41	103	45
– *p*-value	0.030		0.055		0.026	

Data represent numbers of patients, unless indicated otherwise.

At the time when olfactory and/or taste dysfunction was first noticed by the patient, the status of Covid-19 testing was positive in 33 patients, the results were awaiting in 34 patients and 32 patients had not undergone Covid-19 testing. Of the 99 patients with olfactory and/or taste dysfunction, 78 (78.8 per cent) first noticed it after the appearance of other viral symptoms; only 21 patients (21.2 per cent) first noticed it before the appearance of other viral symptoms.

Olfactory dysfunction, taste dysfunction, and olfactory or taste dysfunction were all more common in patients who had malaise or weakness, sore throat, cough, and fever (Table 2), and these differences were statistically significant (all *p* < 0.05).

**Table 2.** General symptoms and olfactory and taste dysfunction

	Olfactory dysfunction		Taste dysfunction		Olfactory or taste dysfunction	
Symptoms	No (*n* = 318)	Yes (*n* = 92)	No (*n* = 318)	Yes (*n* = 92)	No (*n* = 311)	Yes (*n* = 99)
Malaise						
– No	186	13	188	11	185	14
– Yes	132	79	130	81	126	85
– *p*-value	<0.001		<0.001		<0.001	
Sore throat						
– No	258	56	259	55	256	58
– Yes	60	36	59	37	55	41
– *p*-value	<0.001		<0.001		<0.001	
Cough						
– No	259	52	258	53	254	57
– Yes	59	40	60	39	57	42
– *p*-value	<0.001		<0.001		<0.001	
Fever						
– No	237	27	241	23	236	28
– Yes	81	65	77	69	75	71
– *p*-value	<0.001		<0.001		<0.001	

Data represent numbers of patients, unless indicated otherwise.

**Fig. 1.**
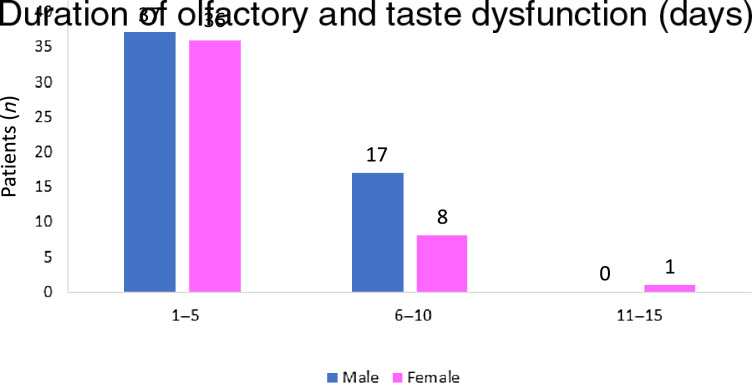
Duration of olfactory and taste dysfunction in coronavirus disease 2019 patients.

The mean duration of olfactory and taste dysfunction was 4.9 days, with a range of 2–15 days (Figure 1). Only in two patients (both females) did olfactory and/or taste dysfunction persist after recovery from Covid-19 infection (after a negative Covid-19 result on reverse transcription polymerase chain reaction testing).

## Discussion

Coronavirus disease 2019 is an active global pandemic; it has so far infected millions and it remains a global threat. As per the initial studies,^1^ the major symptoms associated with Covid-19 included fever, sore throat, cough, fatigue and shortness of breath. Over the past few months, other clinical manifestations have been associated with diagnosed or suspected Covid-19 positivity, such as the sudden appearance of olfactory and taste dysfunction, even in the absence of other symptoms.

The pathophysiology of olfactory and taste dysfunction in Covid-19 infection is still unknown. Chemosensory dysfunction may be seen in some viral infections, and coronavirus is already known to be associated with anosmia.^9^ The SARS-CoV-2 infection most likely causes obstructive inflammation of olfactory clefts, or targets and damages olfactory epithelium support and stem cells, leading to olfactory disturbances.^10^ Some types of coronavirus have been shown to propagate from the nasal epithelium, via the cribriform plate to the olfactory bulb, and the piriform cortex and brainstem.^11^ Direct contact of the virus or its components with gustatory receptors can also lead to taste disturbances. This virus might target angiotensin-converting enzyme 2 (ACE2) expressing cells of the taste buds and/or olfactory epithelium via a cytopathic effect.^12^ Alternatively, an altered neurotransmission in the absence of neurosensory cell death might lead to olfactory and taste dysfunction. Severe acute respiratory syndrome coronavirus-2 may impact the synthesis of neurotransmitters (serotonin and dopamine) by ACE2-expressing cells.^7^

Mao *et al*.^4^ first reported olfactory and taste dysfunction in Covid-19 patients in China in February, 2020. In their retrospective study of 214 laboratory confirmed Covid-19 patients, 11 (5.1 per cent) reported hyposmia and 12 (5.6 per cent) reported hypogeusia. However, these data were derived from medical records, and minor symptoms such as olfactory and taste dysfunction might not have been adequately documented.

A cross-sectional survey was performed by Giacomelli *et al*.^8^ in 59 Covid-19 positive hospitalised patients in Italy. They reported loss of smell or taste in 20 patients (33.9 per cent), and loss of both in 11 (18.6 per cent). Twelve patients (20.3 per cent) reported chemosensory symptoms before hospitalisation. This suggested that olfactory and taste dysfunction could be ancillary symptoms of Covid-19.

Hopkins *et al*.^13^ reported that in 17 per cent of 2428 individuals, new onset anosmia was not associated with other symptoms of Covid-19. In a meta-analysis by Tong *et al*.,^14^ the prevalence of olfactory and gustatory dysfunction was 52.7 per cent and 43.9 per cent respectively.

Lechien *et al*.^7^ studied olfactory and taste dysfunction in 417 mild to moderate laboratory confirmed Covid-19 patients who completed questionnaires, from 12 European hospitals. Olfactory dysfunction was reported by 85.6 per cent of patients, of which 79.6 per cent reported anosmia and 20.4 per cent reported hyposmia. In addition, 12.6 per cent and 32.4 per cent of patients reported phantosmia and parosmia, respectively. Olfactory dysfunction was reported before other symptoms in 11.8 per cent of patients, after other symptoms in 65.4 per cent and at the same time in 22.8 per cent. The olfactory dysfunction persisted after the resolution of other symptoms in 63 per cent of patients.

Kaye *et al*.^15^ used the Covid-19 Anosmia Reporting Tool for Clinicians, developed by the American Academy of Otolaryngology – Head and Neck Surgery, for reporting anosmia and dysgeusia related to Covid-19. On analysis of the first 237 entries, anosmia was noted in 73 per cent of patients prior to a Covid-19 diagnosis and was the initial symptom in 26.6 per cent of patients.

In our study, 22.4 per cent of patients reported olfactory dysfunction, 22.4 per cent reported taste dysfunction, and 24.1 per cent reported either olfactory or taste dysfunction. The differences in the frequency of olfactory and taste dysfunction in various studies may be a result of: possible selection bias, the lack of evaluation of olfactory and taste dysfunction in Covid-19 patients in China at the onset of the pandemic, and potential changes in the virulence and pathogenicity of the virus during the spread of the pandemic.

Lei *et al*.^16^ found differences in the epidemiology and clinical features of patients infected in and outside of Wuhan, and an ethnic pattern. Cao *et al*.^17^ demonstrated a diversity in the expression pattern of ACE2 in Asian and European populations. This is the receptor of SARS-CoV-2, and may lead to differences in the susceptibility and response to this virus from different populations. According to literature, the prevalence of olfactory and taste dysfunction is substantially higher in European Covid-19 patients. However, there have only been a few studies on the occurrence of olfactory and taste dysfunction in Covid-19 patients in Asian patients. The current paper describes a large single-centre study, documenting the occurrence of olfactory and taste dysfunction in Covid-19 patients in India.

• Olfactory and taste dysfunction are significant symptoms in the clinical presentation of coronavirus disease 2019 (Covid-19)
• In this study, 24.1 per cent of patients reported olfactory and/or taste dysfunction
• The study highlights the difference in frequency of olfactory and taste dysfunction occurrence between Asian and European populations
• Knowledge of the spontaneous appearance of olfactory and taste dysfunction and its spontaneous recovery in most patients helps to reassure patients
• There is still a long way to go in understanding the pathogenesis of these Covid-19 associated symptoms

In our study, olfactory and taste dysfunction was proportionally more common in females than males. This finding was similar to that of studies by Lechien *et al*.^7^ and Speth *et al*.^18^ The higher susceptibility of females to develop olfactory and taste dysfunction could be related to gender-related differences in the inflammatory reaction process. In a study by Meini *et al*.,^19^ chemosensory dysfunction in women was less frequent but lasted longer.

Our study showed a significant association between malaise, sore throat, cough, fever and the occurrence of olfactory and taste dysfunction. Lechien *et al*.^7^ also identified a significant association between fever and the occurrence of olfactory and gustatory dysfunction.

In our study, the mean recovery time of olfactory and taste dysfunction was 4.9 days (range, 2–15 days). Meini *et al*.^19^ reported recovery from olfactory and taste dysfunction within four weeks in most patients. Lechien *et al*.^7^ reported an early olfactory recovery rate of 44 per cent, with recovery occurring within the first 8 days following disease resolution in 73 per cent of patients with olfactory dysfunction. Klopfenstein *et al*.^20^ reported a mean duration of anosmia of 9 days, with a complete recovery occurring in almost all patients within 4 weeks. Kaye *et al*.^15^ reported that 27 per cent of their patients experienced at least some improvement of olfactory dysfunction, with a mean time of 7.2 days. Yan *et al*.^21^ reported that 74 per cent of their patients experienced improved olfactory dysfunction with resolving Covid-19 infection.

Most Covid-19 patients with olfactory and taste dysfunction recover their sense of smell and taste within two to four weeks. This short recovery time supports the theory that non-neural olfactory epithelial cells are the potential target of SARS-CoV-2. However, it is possible that stem cells such as horizontal basal cells may be infected, which require more time to recover the olfactory function.^10^ There may be long-lasting olfactory dysfunction if a large percentage of basal cells are damaged and the olfactory epithelium cannot effectively renew over time.^22^

The strength of our study is that it is a prospective study evaluating the occurrence of olfactory and taste dysfunction in Covid-19 patients. It also reports on the association between olfactory and taste dysfunction and other symptoms. This is the first study to report on the frequency of occurrence of olfactory and taste dysfunction in Covid-19 infected patients in India. The paper highlights differences in the frequency of olfactory and taste dysfunction between Asian and European populations.

The limitations of our study are that the patients did not undergo objective evaluation of chemosensory functions, such as psychophysical and/or electrophysiological tests, because of a high risk of Covid-19 exposure to healthcare workers. Secondly, our sample consisted of patients with mild to moderate disease, and they may not be representative of the infected population. The prevalence of olfactory and taste dysfunction in patients with severe disease is difficult to investigate and is unknown. Thirdly, in this study, all symptoms were self-reported by the patients. Therefore, the results of our study should be interpreted with caution. These limitations should be considered in future studies evaluating olfactory and taste dysfunction in Covid-19 patients.

## Competing interests

None declared
